# Access to Burn Care in Low- and Middle-Income Countries: An Assessment of Timeliness, Surgical Capacity, and Affordability in a Regional Referral Hospital in Tanzania

**DOI:** 10.1093/jbcr/irab191

**Published:** 2021-10-13

**Authors:** Matthijs Botman, Thom C C Hendriks, Louise E M de Haas, Grayson S Mtui, Joost Binnerts, Emanuel Q Nuwass, Anuschka S Niemeijer, Mariëlle E H Jaspers, Hay A H Winters, Marianne K Nieuwenhuis, Paul P M van Zuijlen

**Affiliations:** 1 Department of Plastic, Reconstructive and Hand Surgery, Amsterdam UMC, Location VUmc, The Netherlands; 2 Global Surgery Amsterdam, The Netherlands; 3 Amsterdam Public Health Research Institute, The Netherlands; 4 Department of Surgery, Haydom Lutheran Hospital, Tanzania; 5 Association of Dutch Burn Centers, Burn Centre Martini Hospital, Groningen, The Netherlands; 6 Hanze University of Applied Sciences, Groningen, The Netherlands; 7 Burn Center and Department of Plastic, Reconstructive and Hand Surgery, Red Cross Hospital, Beverwijk, The Netherlands; 8 Pediatric Surgical Centre, Emma Children’s Hospital, Amsterdam UMC, University of Amsterdam, Vrije Universiteit, The Netherlands

## Abstract

This study investigates patients’ access to surgical care for burns in a low- and middle-income setting by studying timeliness, surgical capacity, and affordability. A survey was conducted in a regional referral hospital in Manyara, Tanzania. In total, 67 patients were included. To obtain information on burn victims in need of surgical care, irrespective of time lapsed from the burn injury, both patients with burn wounds and patients with contractures were included. Information provided by patients and/or caregivers was supplemented with data from patient files and interviews with hospital administration and physicians. In the burn wound group, 50% reached a facility within 24 hours after the injury. Referrals from other health facilities to the regional referral hospital were made within 3 weeks for 74% in this group. Of contracture patients, 74% had sought healthcare after the acute burn injury. Of the same group, only 4% had been treated with skin grafts beforehand, and 70% never received surgical care or a referral. Together, both groups indicated that lack of trust, surgical capacity, and referral timeliness were important factors negatively affecting patient access to surgical care. Accounting for hospital fees indicated patients routinely exceeded the catastrophic expenditure threshold. It was determined that healthcare for burn victims is without financial risk protection. We recommend strengthening burn care and reconstructive surgical programs in similar settings, using a more comprehensive health systems approach to identify and address both medical and socioeconomic factors that determine patient mortality and disability.

Every year, nearly 11 million people globally suffer from burns that require medical attention, ranking it fourth among all injuries.^1^ In low- and middle-income countries (LMICs), fire-related burns are among the leading causes of disability and life years lost, with children as the most affected age group.^1,2^ Worldwide, the mean burden of child burn deaths is 2.5 per 100,000 across 103 countries, with the largest burden in sub-Saharan Africa (4.5 per 100,000).^3^ In a review of burns in sub-Saharan Africa, children aged 10 years and younger represent more than 80% of the burn patient population.^4^

Since 2015, a team of physicians from the Netherlands has collaborated in Tanzania with physicians from Haydom Lutheran Hospital (HLH), a regional referral hospital in the Manyara region. As requested by the Tanzanian doctors, the main goal of the collaboration is to improve burn care and reconstructive surgical skills, by organizing twice-yearly surgical training camps that focus on acute burn management and burn contracture release surgery.

The safety and surgical capacity in the hospital were evaluated between 2017 and 2018. Analysis of capacity showed that the surgical care provided was safe and effective for patients with severe burn wounds or burn contractures.^5–7^ However, the data also showed that despite the Tanzanian and Dutch training program, there was high mortality among burn victims arriving late to the hospital, as well as a high rate of disabled burn contracture patients, mostly only coming to the hospital during the twice-yearly reconstructive surgery training camps.

Based on these observations, two questions were raised: 1) What factors contribute to delayed arrival of acute burn wound patients? 2) Why are burn injuries that occur in the catchment area of the hospital still developing into severe contractures?

Prior to data collection, the leading assumption was that geographical and socioeconomic factors, cultural beliefs, and/or barriers related to traditional notions of illness and healing could be attributable to poor burn management at nearby healthcare facilities. Essentially, social and economic barriers persisting for patients in the area despite the provision of adequate and safe burn care at the regional referral hospital. If so, then these barriers may involve factors that delay and/or hamper patients’ access to burn care.

Access to healthcare stems from a complex interaction of factors between patients’ health-seeking behaviors and healthcare provision. The interplay of both has been defined as “the opportunity to reach and obtain appropriate healthcare services in situations of perceived need for care.” ^8^ In 2015, Alkire et al^9^ studied access to surgery in 180 countries using four criteria: timeliness, safety, affordability, and surgical capacity. Based on their probability models, 4.8 billion people, which is 68% of the world’s population, lack access to safe and affordable surgical care, most of them living in LMICs.

Little information is available on access to care for burn victims in LMICs. The limited data available is from studies in Nigeria, Ethiopia, Malawi, and Ghana. These studies suggest that only a few patients can access burn care in a timely fashion; that few patients know the potential consequences of delayed burn care; and that resource-constrained populations are at risk of inaccessibility.^2,10,11^ Therefore, more detailed information on access to burn care in LMICs is needed to improve the current situation.

Gaining more detailed information on access to burn care in LMICs is the aim of this article. The data presented assess access to burn care in terms of timeliness, surgical capacity, and affordability in a regional referral hospital in Manyara, Tanzania. The obtained insights, in turn, can be used to reduce barriers to accessing burn care in LMICs. Doing so, the article contributes to the goal of improving burn-related mortality and disability, where most burn injuries occur, globally.

## METHODS

An in-hospital survey was conducted at HLH, which services an area estimated to have between 2 and 7 million people. There is no official burn center in the area yet, and in this hospital, burn care is provided by teams of nurses, doctors, physiotherapists, and medical specialists (surgeons and pediatricians) that have been trained in burn care and reconstructive surgery by visiting plastic surgeons from the Netherlands.

Patients were included in the program—research and surgical training—over three different periods during the months that the training camps took place. The first period was October 2017, the second was June 2018, and the third was October 2018. We identified and enrolled patients presenting with severe burns. We estimate that of the severe burn and contracture patients, consulting HLH within these periods, 80% were included in this study.

### Participants

Two groups of patients were eligible:

1. Patients of all ages with severe burns, defined as partial-thickness burns greater than 10% of the total body surface area (TBSA): burns to the face, hands, feet, genitals, perineum, or across major joints; and any full-thickness burns.^12^
2. Patients of all ages with contractures of joints after burn injury in need of contracture release surgery.

All patients provided informed consent.

### Ethical Clearance

Ethical clearance approval was obtained from the National Institute for Medical Research in Tanzania (NIMR/HQ/R.8a/Vol.IX/2652). Written informed consent was obtained from all participants, but if a participant was functionally illiterate, a thumbprint with an additional signature from a literate witness was obtained. For individuals younger than 18 years, a parent, caregiver, or guardian provided written consent.

### Access to Burn Care Survey

To assess access to burn care, a survey was developed based on the Surgeons Overseas Assessment of Surgical need population-based survey.^13^ Questions were modified in line with participant observations. The survey was divided into four parts: basic characteristics, timeliness, surgical capacity, and affordability.

Differences in patient groups mandated two versions of the survey: one for the burn wound group (Supplementary Appendix 1) and one for the contractures group (Supplementary Appendix 2). Each version consisted of 30 questions and was completed after admission. The surveys were conducted in Swahili, Iraqw, or English. Language assistance was provided by translators. For pediatric patients, surveys were administered to caregivers.

### Basic Characteristics and Socioeconomic Factors

The following basic characteristics were collected: age, sex, etiology, TBSA affected, and maximum depth of the burn wound. Economic factors were interpreted based on levels of literacy and education and occupation, and sociocultural information focused on learning the patients’ tribe. For children younger than 18, the primary earner of the household disclosed the highest level of education.

### Timeliness

Timeliness is defined as the physical ability to reach a healthcare facility and the health-seeking behavior of the patient and the caregiver.^14^ Regarding geographical accessibility, time between the accident and presentation at the first healthcare facility, mode of transport, waiting time for transport, and total transport time to the hospital were collected. Transport data for burn wound patients consisted of transport data to HLH, or if Haydom was not the first healthcare facility reached, then transport data to the first healthcare facility and transport data from this facility to HLH was collected. For contracture patients, transport from home to HLH was investigated. Participants who arrived more than 24 hours after the burn accident took place were asked to share their reasons for being delayed. Answers were categorized into geographical barriers, lack of trust, lack of money, and health-seeking beliefs.

### Surgical Capacity

With respect to both the groups, data were collected on:

(1) Type of facility, other than HLH, first consulted after the initial burn injury (traditional healer, clinic/dispensary/hospital/referral hospital).
(2) Treatment provided during initial presentation elsewhere (eg, conservative treatment, skin grafting, and amputation).
(3) Days between initial presentation elsewhere and referral to HLH.
(4) Types of surgical procedures, as part of treatment, provided at HLH.
(5) Reasons for not receiving surgical care at HLH, when indicated otherwise.

### Affordability

Regarding affordability, data were collected on the patient’s health insurance coverage. This included national health insurance covering most primary and secondary healthcare services, and community health insurance covering primary healthcare services, including emergency surgery and a 5-day hospital stay.

Data were also collected on a daily available budget (in Tanzanian shillings) per head of household. The amount was converted to U.S. dollars, using the official exchange rate of the World Bank (July 2019). In addition, patients’ hospital fee information was retrieved from the financial department of the HLH, which included the total fee covered by the patient 1 month after the data collection period (July 2019) and the outstanding amount at that moment.

*Catastrophic healthcare expenditure* has been defined in previous studies as an out-of-pocket cost equal to or greater than 10% of an individual’s yearly expenditure.^15^ This definition was used to calculate the percentage of patients facing a catastrophic expenditure in the study cohort. HLH has the capacity to use a cost-sharing model to protect patients from catastrophic healthcare expenditure. This means that the treatment costs can be shared between the hospital and patients. Yet to determine whether or not to subsidize patients’ costs, patients are first referred to HLH’s Social Welfare department and undergo a counseling process, led by social healthcare workers who consult with patients’ respective family and village leaders. Only after consensus between all parties, do patients then receive an offer for a payment arrangement, in which costs can be paid over installments.

The survey data were supplemented with quantitative information supplied by the hospital administration, including treatments provided, which were then compared with hospital bills and payments already received. Additional qualitative data were obtained through participant observation and through discussion groups, evaluation meetings of the training program, and unstructured interviews with patients’ immediate healthcare providers.

### Statistical Analyses

For dichotomous parameters, calculated percentages per group were used. Differences between groups were tested with chi-squared tests. To describe differences between groups, use of means (*SD*) and *t* tests if variables were normally distributed, medians and interquartile ranges (IQRs) and Mann–Whitney *U* tests if data were measured at ordinal level or not normally distributed. Data were analyzed with SPSS version 25. An alpha of 5% was adopted.

## RESULTS

### Basic Characteristics and Socioeconomic Factors

The surveys were completed by all eligible patients or their caregivers: 36 patients in the burn wound group and 31 patients in the contracture group (Table 1). Most of the patients were children, defined as research participants younger than the age of 18 years (86% in the burn group, and 84% in the contracture group, ns *P* = .82). In these cases, the survey questions were answered by the caregivers. The median age was 4 years (IQR 2–9, range 0.5–48) in the burn wound group and 6 years (IQR 4–12, range 2–44) in the contracture group. Among burn wound patients, 53% of the burns were scalds. Among contracture patients, fire burns were the most common (65%). This difference is not significant (*P* = .32). Medical histories of the participants indicated that their burn injuries were occurring almost exclusively around areas with open fire for cooking and heating water. The median TBSA in the burn wound group was 10% (IQR 7–18). In the contracture group, it was estimated to be 4% (IQR 2–9), with fingers as the most common location for the contractures.

**Table 1.** Basic characteristics

	Acute Burns	Contractures
Total number of patients, N (%)	36 (100)	31 (100)
Females, N (%)	18 (50)	20 (65)
Males, N (%)	18 (50)	11 (35)
Age, median years (IQR, range)	4 (2–9, 0.5–48)	6 (4–12, 2–44)
*Etiology (N, %)*		
Scalds	19 (53)	11 (35)
Fire	14 (39)	20 (65)
Contact	3 (8)	0 (0)
Electricity	0 (0)	0 (0)
*Burn characteristics*		
TBSA, median % (IQR)	10 (7–18)	4 (2–9)
*Depth (N, %)*		
Superficial	0 (0)	NA
Superficial partial-thickness	6 (17)	NA
Deep partial-thickness	9 (25)	NA
Full-thickness	18 (50)	NA
Deeper injury	3 (8)	NA

*IQR*, interquartile range.

For the burn group, 97% of the caregivers (for children) or patients (adults) reported to have no formal education or only reached primary school level. This number was at 84% for the contracture group (Table 2). Patients and patients’ caregivers were primarily subsistence-based farmers (ie, families that farm for their own living with limited landownership, 67% in the burn wounds group vs 65% in the contracture group, *P* = .86).

**Table 2.** Socioeconomic factors

	Acute Burns	Contractures
*Education, N (%)*		
None	5 (14)	7 (23)
Primary education	30 (83)	19 (61)
Secondary	1 (3)	3 (10)
Tertiary	0 (0)	2 (6)
*Literacy, N (%)*		
Yes	30 (83)	24 (77)
None	6 (17)	7 (23)
*Occupation, N (%)*		
Unemployed	2 (5)	4 (13)
Domestic helper	2 (5)	0 (0)
Farmer	24 (67)	20 (65)
Shop owner/self-employed	5 (14)	4 (13)
Government employee	1 (3)	0 (0)
Nongovernment employee	2 (5)	1 (3)
Studying	0 (0)	2 (6)
*Tribe, N (%)*		
Iraqw	18 (50)	16 (52)
Datooga	8 (22)	5 (16)
Nyiramba	4 (11)	2 (6)
Nyaturu	2 (6)	1 (3)
Sukuma	1 (3)	0 (0)
Ngoni	0 (0)	0 (0)
Maasai	1 (3)	0 (0)
Makonde	1 (3)	0 (0)
Nyakyusa	1 (3)	0 (0)
Pare	0 (0)	2 (6)
Chagga	0 (0)	1 (3)
Rangi	0 (0)	1 (3)
Gogo	0 (0)	2 (6)
Zigula	0 (0)	1 (3)

### Timeliness

Many patients from the burn wound group reached their first healthcare facility within one day (median 1, IQR 0–1 days) (Table 3). That was also the case for patients who came directly to HLH (median 1 day, IQR 1–6 days). Seven patients did not visit a healthcare facility within 24 hours; arriving at the first healthcare facility, up to 49 days after the burn injury. The reasons given were lack of trust and/or money. No differences were found in basic characteristics, socioeconomic factors, or the travel times of these 7 patients when compared to the group that did reach the facility within 24 hours. The median time for patients to reach Haydom after visiting another healthcare facility was 3 days (IQR 1–8). Eleven burn patients (33%) visited a traditional healer first, and for this group, it also took a median of 3 days before coming to Haydom (IQR 1–13). Overall, 18 (50%) of the burn wound patients reached Haydom within 24 hours. The median traveling time to reach the first healthcare facility was 1 hour (IQR 0.4–3.0). Waiting time for transport was half an hour (median, IQR 0.15–1.0). (See Table 3 for details on the transportation component of the study.)

**Table 3.** Timeliness

	Acute Burns	Contractures
*Timing of presentation, median days (IQR)*		
Time between burn injury and presentation at any healthcare facility	1 (0–1)	NA
Time between burn injury and presentation at HLH	1 (1–6)	650 (410–1566)
Time between consultation of the first healthcare facility and arrival at HLH	3 (1–8)	NA
*Patients that were admitted at HLH within 24 hours, N (%)*	18 (50)	NA
*Main reason for not consulting a health facility within 24 hours*		
Total patients, N (%)	7 (19%)	NA
Believed that it would heal without hospital care	1	NA
No money for healthcare or transport	3	NA
No trust/fear	3	NA
*Mode of transport to the first healthcare facility (multiple answers possible), N*		
Ambulance	4	4
Bus or public landcruiser	11	11
Taxi	10	3
Motorbike taxi	10	9
Private motorbike	3	1
Bicycle	1	1
On foot	7	4
*Transport time to reach first healthcare facility, median (IQR)*		
Hours of traveling	1 (0.5–3.0)	NA
Hours waiting time for transport	0.5 (0.15–1.0)	NA
*Transport time to reach HLH for contracture patients, mean (IQR)*		
Hours of traveling	Not known	2 (0.5–4)
Hours waiting time for transport	Not known	0.5 (0–1.75)

*IQR*, interquartile range; *HLH*, Haydom Lutheran Hospital.

For the contracture group, despite participants’ difficulties remembering how long it took to reach their first healthcare facility after the burn injury, the median time between the injury and presenting their contractures at HLH was 650 days (median, IQR 410–1566 days). The median travel time to HLH was 2 hours (IQR 0.5–4.0 hours), and the median waiting time for transport was half an hour (IQR 0–1.75 hours).

### Surgical Capacity

Nineteen burn wound patients (53%) consulted another healthcare facility (a dispensary, health center, or a District hospital) before coming to HLH (Table 4). The healthcare provided at these other facilities was conservative wound care in 95%, with patients’ reported treatment frequently lacking consistency in the use of antibiotics, pain medication, fluid resuscitation, and/or dressing. Only one patient was treated surgically in another facility, for a debridement without skin grafting.

**Table 4.** Initial burn care

	Acute Burns	Contractures
Patients who consulted another healthcare facility first, N (%)	19 (53)	23 (74)
Patients who consulted HLH as first facility, N (%)	17 (47)	8 (26)
*Other healthcare consulted (multiple answers possible), N*		
Traditional healer	11	8
Dispensary or health center	8	8
District hospital	11	22
*Type of treatment received since burn injury at other healthcare facilities, N (%)*		
Total number of patients who received treatment	19 (100)	23 (100)
A conservative treatment (IV fluids, pain medication, antibiotics, and/ or dressings)	18 (95)	23 (100)
Surgical debridement	1 (5)	3 (12)
Amputation	0 (0)	0 (0)
Skin grafting	0 (0)	1 (4)
*Reasons for not receiving surgical treatment at other healthcare facilities, N (%)*		
Total patients who did not receive any form of surgery before	35 (97)	29 (94)
Believed that it may heal without surgery	1	1
No money for healthcare or transport	0	8
No trust/fear	0	4
It was not available elsewhere according to the patient or caregiver	34	16
*Referral from other facility to HLH, N (%)*		
Referral indicated (defined as: need for skin grafting and/or contracture release)	19 (100)	23 (100)
Referral (and arrived to HLH within 3 weeks)	14 (74)	NA
Not referred according to the patient/caregiver	2 (10)	16 (70)
The patient did not follow-up referral within 3 weeks	3 (16)	NA

*HLH*, Haydom Lutheran Hospital.

All 19 burn wound patients who had previously consulted another healthcare facility had an indication for surgical care when arriving in Haydom later. In this group, 14 patients (74%) indicated that they were referred within 3 weeks from the day of the injury. Two patients indicated that they were not referred at all, and 3 patients reported being referred in time, but waited a long time before consulting HLH out of a lack of trust and insufficient funds.

In the contracture group, 23 patients (74%) indicated that they had visited a healthcare facility before for their burn injury. Among these patients, only 4 (17%) received previous surgical treatment at that time: Three (13%) had been treated with only surgical debridement and 1 patient (4%) with skin grafting before coming to Haydom. Sixteen (70%) of the contracture patients who consulted another healthcare facility after the accident indicated that they were never referred to a hospital that provides surgical burn care or burn contracture release surgery.

At the time of presentation at HLH, surgical care was indicated in 27 patients (75%) of the burn group and all contracture patients. All contracture patients had an indication for contracture release and received surgical care. However, in the burn group, 7 patients (26%) with an indication for surgery did not receive surgical care although it was available. The reasons given were lack of trust in the surgical techniques (5 patients) and lack of money (2 patients). The mean length of hospital stay was 38 days for the burn patients and 11 days for the contracture patients. The most common procedure for the burn group was delayed skin grafting after surgical or spontaneous debridement (51%). Different techniques were used for contracture release surgery, including full-thickness grafts and local flaps (Table 5).

**Table 5.** Treatment at HLH

	Acute Burns		Contractures
Only conservative treatment indicated, N (%)	9 (25)		0 (0)
Surgery indicated, N (%)	27 (75)		31 (100)
Surgery provided, N (%)	20 (55)		31 (100)
*Length of stay at HLH, mean days (range)*	38 (2–203)		11 (3–28)
*Reason for not receiving surgery when indicated at HLH*			
Total number of patients that did not receive surgical care	7 (100)		NA
No money for healthcare	2 (29)		
No time	0 (0)		
No trust/fear	5 (71)		
No care available at HLH	0 (0)		
*Surgical procedures (multiple answers possible)*			
Total number of procedures, N (%)	39 (100)	Total number of procedures	31 (100)
Escharotomy	3 (8)	Total number of techniques	60 (100)
Amputation	0 (0)	Five flap plasty	9 (15)
Only debridement	14 (36)	Classic Z-plasty	14 (23)
Early debridement with skin grafting	2 (5)	Interposition flap	7 (12)
Delayed skin grafting	20 (51)	Full thickness skin graft	24 (40)
		Split thickness skin graft	6 (10)

*HLH*, Haydom Lutheran Hospital.

### Affordability

The results on affordability show that 25% of the burn patients and 26% of the contracture patients had their costs covered by health insurance (Table 6). The mean treatment fee was $378 per patient for the burn group and $167 per patient for the contracture group. The longer hospital stays and the cost of daily wound dressing in the burn group contributed to the difference. The mean daily budget of families of the patients was $0.73 (*SD* 0.68) for burn patients and $0.70 (*SD* 0.62) for contracture patients.

**Table 6.** Affordability

	Acute Burns	Contractures
*Insurance (N, %)*		
National health insurance (full insurance)	1 (3)	4 (13)
Community health insurance (first 5 days of admission)	8 (22)	4 (13)
No Insurance	27 (75)	23 (74)
*Budget in U.S. $, mean (SD)*		
Daily budget per household	0.73 (0.68)	0.70 (0.62)
*Patient fee in U.S. $, mean (SD)*		
Hospital fee	378.80 (419.51)	166.70 (97.55)
Covered by patients/insurance	170.59 (140.75)	104.30 (104.90)
Outstanding amount of all patients 3 months after discharge of the last patient	208.19 (375.02)	62.40 (85.80)
*Transportation costs*		
Costs in U.S. dollars, mean	11.90	5.40

Given these figures, the hospital patient fees exceeded the catastrophic health expenditure threshold by up to 6 times for the contracture groups and up to 15 times for the acute burn wounds group.

For patients with limited resources, payments were accepted in small installments over a longer period. However, most patients still could not accomplish micropayments. At the end of the data collection period of this study (June 2019), it was decided to cancel the outstanding debt for all patients—a mean $171 per patient (ie, 45% paid). For the contracture group, the mean amount covered was $104, corresponding to 63% of the total fee.

### Qualitative Results

Participant observation during the treatment and during patient follow-up identified patients’ and caregivers’ beliefs and fears. Traditional means for treating illness and injury reported the application of ash and eggs on the burn wounds; a treatment perceived by patients and caregivers to be the preferred first step in burn treatment. The patients expressed that the hospital in Haydom was trusted for its medical care, but fear of costs for treatment was common. This was not without reason. During observations and patient outreach activities for follow-up of up to 2 years after the injury, it became apparent that debts faced, even after accepting payments in installments, became a large burden for families involved. It was not uncommon for half of the families, who owned land and animals, to sell these assets, as well as rely on neighbors to help feed their children.

In Haydom, skin grafting was available during the entire inclusion period of the research. Despite availability, 7 burn patients did not receive grafting surgery when indicated. Lack of patient trust was identified as the cause during discussion groups with Haydom physicians. Skin grafting was primarily performed with a Humby knife, which malfunctioned. This caused deep donor site wounds with delayed healing. Haydom physicians speculated that these experiences may have resulted in patients’ fears or resistance to skin grafting techniques. To counter complications resulting from the Humby knife, the surgical training program included a donated electric dermatome. Access to this technology resulted in fewer donor site complications. However, it was observed that fears of the skin grafts were slow to dissipate. Taking the qualitative information into consideration indicates the importance of the presence, handling, and maintenance of essential surgical tools and provides additional insight into the effect of complications on patients’ confidence in treatment methods.

## DISCUSSION

The aim of this study was to assess potential barriers against access to burn care in an LMIC. Despite a twice-yearly surgical training program to improve skills and equipment for burn care, after 2 years, the lack of timeliness, surgical capacity, and affordability were still found to be important barriers for burn patients in the HLH’s catchment area.

First, timeliness in reaching a healthcare facility, where adequate burn care can be provided, is clearly a factor that needs improvement for patients with severe burns in need of emergency care. Although arriving in a healthcare facility within 24 hours was achieved by 80% of the burn patients, only half of the burn patients reached a facility providing surgical burn care within 24 hours.

Second, survey responses on surgical capacity indicate that other healthcare facilities, including several district-level hospitals, rarely provide surgical treatment for burn patients. This finding is consistent with the literature on surgical burn care in sub-Saharan Africa showing a high need for improvement of surgical burn care in this setting.^4,16^ When surgical burn care is unavailable at the first health facility, then referral knowledge is key for a good outcome. Within the burn wounds group, 74% of patients who went to another facility first indicated receiving a referral to HLH. In the contracture group, only 30% were referred to HLH and if so, only for the contracture release, and not earlier after the acute burn injury happened. Although we can clearly state that all contracture patients must have had an indication for skin grafting after their burn injury, only 4% in this group had been treated with a skin graft previously. One solution has been to offer community outreach opportunities. This will be done through a local HLH burn care awareness day.

Third, surgical care was unaffordable for the majority of patients in the study, regardless of a cost-sharing model at HLH. This cost-sharing model keeps patients’ fees at an average of $378 per patient, per admission is comparable with other low-cost initiatives to provide burn care in similar settings.^17^ However, the mean income of $0.73 per day implies that any hospital fee that exceeds $26 would already be a catastrophic expenditure for the majority of patients’ families.^15^ However, for the contractures group, the mean hospital bill was 6 times higher, and for the burn wounds group, it was 15 times the catastrophic expenditure threshold. It is important to understand that this calculation does not include indirect costs. It is also important to realize that due to previous expenditures during the acute phase of the burn wound, the contracture patients face a second financial burden. As a local solution, financial support from the visiting team from the Netherlands provides an ad-hoc solution to cover the patients’ costs, but this may not be the most sustainable approach. In collaboration with the team at HLH, plans are in place to organize local and international fundraising events. Perhaps, this is one step to improving the local situation; however, more action is needed on a larger scale. In sub-Saharan Africa, where burn injuries are very common, structural support is lacking. If families must sell their means of livelihood to pay for the healthcare for a burn victim, how willing will other families in the same situation be to accept future medical interventions? Financial risk protection requires a health system approach to achieve universal health coverage in 2030.^18^ The rise in coverage from the national health insurance in Tanzania over the past years is a promising sign, but the national health insurance coverage is still less than 10%.^19^ Strengthening financial risk protection strategies is needed to improve timely access to healthcare, including burn care, in the coming years. Additionally, this wider health system strengthening approach should focus on creating and distributing subsidized prevention strategies around improving the cooking conditions and promoting safer at-home methods of fire prevention, which are locally inspired. Guidelines from international experts and organizations, like the standards of Interburns and the Netherlands Burn Foundation, could become available to assist in these strategies to improve burn care in similar settings.^20^

### Limitations

This study has several limitations. Timeliness, surgical capacity, and affordability were assessed using a survey and by collecting data from the HLH administration and patient files. Thereby, all data were obtained from patients who came, early or late after the burn injury, to HLH. This selection bias prevents us from extrapolating the results of this study to the patients in the catchment area who never went to HLH for healthcare. The researchers could also not provide absolute confirmation that skin grafting was seldom performed elsewhere because of the scale of the study’s design. However, the lack of skin grafting in the area can be inferred by commentaries from healthcare workers at other facilities—for example, statements that assert that a well-functioning dermatome was not available in many of the health facilities.

While this study indicates that affordability is a primary concern for patients leading to delays in access to burn care, secondary factors may exist. These factors, which were discussed with Haydom physicians, likely involve localized traditional beliefs and social obligations to traditional healing, which deserve greater attention than this study could accommodate.

Surveyed answers provided by caregivers must be interpreted due to gaps in the levels of education between study participants and investigators, trust issues with clinicians, and perceived personal gain in relation to optimal survey responses. Additionally, this study accounts for unintentional researcher bias, as the investigators were part of the treatment team. Setting and sponsor biases must also be considered, due to the context in which patients were surveyed within the HLH care environment, possibly prompting them to respond in a specific manner that could optimize personal healthcare outcomes.

## CONCLUSION

This study identified timeliness. lack of trust and adherence to traditional beliefs, surgical capacity, and affordability as important barriers to accessing burn care in rural Tanzania.

To assure timely, safe, and affordable burn care in similar settings in LMICs, access to adequate care needs to be improved. We recommend strengthening burn care with a comprehensive approach that goes well beyond a single hospital management approach. And is instead an approach that may benefit from support from foreign experts, while continuing to have local ownership and be coordinated by regional and national bodies. The roles for actors within the healthcare system to support this strategy should include improving health-seeking action, such as raising awareness in local communities, and reinforcing knowledge and skills of healthcare providers by including all facility levels in a health catchment area to assure emergency burn care, stabilization, and timely referral. This wider approach should also include subsidized financial risk protection. The lack of affordable treatment options for patients in need undermines all efforts to improve quality of care. Future initiatives should go hand in hand with new research projects, needed to identify and address both medical and socioeconomic factors to tailor the comprehensive health system approach to the local needs, to effectively reduce burn-related mortality and disability in LMICs settings.

## Supplementary Material

irab191_suppl_Supplementary_Appendix_1

irab191_suppl_Supplementary_Appendix_2

## Notes

Funding and Conflict of Interest: All authors declare they have no conflicts of interest nor did they receive funding for the work from any source.
