# Supplementary material for: Access to Burn Care in Low- and Middle-Income Countries: An Assessment of Timeliness, Surgical Capacity, and Affordability in a Regional Referral Hospital in Tanzania
Source: J Burn Care Res. 2021 Oct 13;43(3):657–64. doi: 10.1093/jbcr/irab191 (PMC9113785; doi:10.1093/jbcr/irab191)
Supplement: irab191_suppl_Supplementary_Appendix_1 [file irab191_suppl_supplementary_appendix_1.pdf]

Global
Surgery
Amsterdam

ASSESSMENT OF ACCESS TO BURN CARE
acute burn wounds

Questions are answered by

☐Patient

☐Caregiver – Relation to the patient: ______________

1 PATIENT DEMOGRAPHICS

1A Gender:

☐Male

☐Female

1B Age:

Year of birth _________

1C Address:

Kijiji:

____

Kata:

____

Wilaya:

____

Mkua:

____

1D Education:
What is the highest educational level that you have achieved or are currently following?

☐None (includes nursery)

☐Primary school

☐Secondary school (junior / senior)

☐Tertiary (diploma, colleges, bachelors)

☐Graduate degree (Master degree, PhD)

1E Literacy:
Are you able to read and write in any language?
[For adults and children who are currently learning how to read and write answer: 'No']

☐Yes

☐No

1F Occupation:
What is your primary occupation?

☐Unemployed [Currently looking for jobs, retiree's, students]

☐Home maker [Housewives]

☐Domestic helpers [Cleaners, housekeepers, watch guards]

☐Farmer [Herders, agriculture, pastoralist]

☐Self-employed / small-business [Small business owners like: shops, kiosks, food traders]

☐Government employee [Police officer, accountant, teachers, health care workers]

☐Non-government employee [Cooperation managers, NGO-staff]

1G Tribe
What is your ethnic background?
[In case of refusal to answer, ask whether the person was born in Tanzania]

2. INJURY

2.A TBSA (%)

______%

2.B Depth (maximum)

☐ Superficial burn wound (first degree)

☐ Superficial dermal burn wound (second degree)

☐ Deep dermal burn wound (deep second degree)

☐ Full thickness burn wound (third degree)

☐ Full thickness burn wound involving under lying structures (fourth degree)

3. TIMELINESS

3A Transport to HLH

What is the main way for you to go to HLH?

☐Public transport (bus/taxi)

☐Private car

☐Private motorcycle

☐Pikipiki

☐Bicycle

☐Animal

☐On foot

☐Carried

☐Ambulance

3B Hours of traveling from home to HLH:

How long does it take you in total to get to HLH if you don't have to wait for transportation?

______ hours

3C Waiting time for transport:

How long do you probably have to wait for transportation?

______ hours

3D KM of traveling from home to HLH:
How many kilometers do you have to travel to get from home to HLH?

______ KM

3E Time of injury *, time of admission *, duration of admission*:

Date of injury:

____

Date of presentation at first healthcare facility:

____

Length of admission first healthcare facility:

____

Date of admission to HLH:

____

Time injury to admission/presentation HLH:

____

Duration of admission HLH:

____

4. SURGICAL CAPACITY

4A Healthcare received before admission to HLH:

What kind of treatment did you receive before you came to HLH?

☐None

☐Traditional healer

☐Dispensary

☐Primary healthcare facility

☐Hospital

4A.1 Reasons for not seeking burn care (>3 days after the injury):

☐No need

☐No money for health care

☐No (money for) transportation

☐No time

☐Fear/no trust

4A.2 If treated by another hospital/healthcare facility, what kind of treatment was received?

☐Conservative treatment (wound care/injections/oral medication)

☐Escharotomy

☐Escharectomy

☐Escharectomy and early skin graft

☐Early skin graft

☐Delayed skin graft

☐Amputation

☐Muscle flap

4B. Referral by other hospital/healthcare facility:

Was referral to HLH indicated?

☐Yes

☐No

4B.1 Were you referred in an acceptable time (before 2-3 weeks)?
[Only when "traditional healer" is answered to 4A]

☐Yes

☐No

4B.2 What was the reason for delay in referral?

☐Not referred because not informed by other hospital/healthcare facility

☐I refused referral

4C Was surgical care indicated?

☐Yes

☐No

4C.1 Did you receive surgical care at HLH?

☐Yes

☐No

4C.2 If yes, what kind of surgical care did you receive and how often?
[Only when "yes" is answered to 4B]

☐ Conservative treatment (wound care/injections/oral medication)

☐Escharotomy

☐Escharectomy

☐Escharectomy and early skin graft

☐Early skin graft

☐Delayed skin graft

☐Amputation

☐Muscle flap

4C.3 If no, what were the reasons for not receiving surgical care?
[Only when "no" is answered to 4B]

☐No need

☐No money for health care

☐No (money for) transportation

☐No time

☐Fear/no trust

☐Not available (facility/personnel/equipment)

5. AFFORDABILITY

5A Insurance:
Do you have an insurance?*

☐NHIF

☐CHF

☐Other

☐None

5B Daily budget
What is the daily budget of the household?

_______ Tanzanian shilling per day

☐Tsh 0 – Tsh 4500 per day

☐Tsh 4500 – Tsh 6000 per day

☐Tsh 8000 – Tsh 10,000 per day

☐Tsh 10,000 – Tsh 15,000 per day

☐Tsh 15,000 – Tsh 25,000 per day

☐Tsh 25,000 – Tsh 50,000 per day

☐Tsh >50,000 per day

5C Acres of land

How many acres of land do you own?

_______ acres

5D Amount of patient fee

Total amount of patient fee

_______ Tanzanian shilling

Fee covered by patient

_______ Tanzanian shilling

What was arranged about the outstanding amount of money?

☐The amount was to be paid in installments

☐The amount was absconed

☐The amount was paid by a poor patient fund

5D Transportation costs:

What does it cost you to travel one time from home to HLH?

_______ Tanzanian shilling
