# Supplementary material for: Access to Burn Care in Low- and Middle-Income Countries: An Assessment of Timeliness, Surgical Capacity, and Affordability in a Regional Referral Hospital in Tanzania
Source: J Burn Care Res. 2021 Oct 13;43(3):657–64. doi: 10.1093/jbcr/irab191 (PMC9113785; doi:10.1093/jbcr/irab191)
Supplement: irab191_suppl_Supplementary_Appendix_2 [file irab191_suppl_supplementary_appendix_2.pdf]

ASSESSMENT OF ACCESS TO BURN CARE
contractures

Questions are answered by

☐Patient

☐Caregiver – Relation to the patient: _______________

1 PATIENT DEMOGRAPHICS

1A Gender:

☐Male

☐Female

1B Age:

Year of birth __________

1C Adress:

Kijiji:

____

Kata:

____

Wilaya:

____

Mkua:

1D Education:
What is the highest educational level that you have achieved or are currently following?

☐None (includes nursery)

☐Primary school

☐Secondary school (junior / senior)

☐Tertiary (diploma, colleges, bachelors)

☐Graduate degree (Master degree, PhD)

1E Literacy:
Are you able to read and write in any language?
[For adults and children who are currently learning how to read and write answer: 'No']

☐Yes

☐No

1F Occupation:
What is your primary occupation?

☐Unemployed [Currently looking for jobs, retiree's, students]

☐Home maker [Housewives]

☐Domestic helpers [Cleaners, housekeepers, watch guards]

☐Farmer [Herders, agriculture, pastoralist]

☐Self-employed / small-business [Small business owners like: shops, kiosks, food traders]

☐Government employee [Police officer, accountant, teachers, health care workers]

☐Non-government employee [Cooperation managers, NGO-staff]

1G Tribe
What is your ethnic background?
[In case of refusal to answer, ask whether the person was born in Tanzania]

____

2. INJURY

2.A Estimated TBSA (%)

_______%

2.B Location

____

3. TIMELINESS

3A Transport to HLH

What is the main way for you to go to HLH?

☐Public transport (bus/taxi)

☐Private car

☐Private motorcycle

☐Pikipiki

☐Tuktuk

☐Bicycle

☐Animal

☐On foot

☐Carried

☐Ambulance

3B Hours of traveling from home to HLH:

How long does it take you in total to get to HLH if you don't have to wait for transportation?

______ hours

3C Waiting time for transport:

How long do you probably have to wait for transportation?

_______ hours

3D KM of traveling from home to HLH:
How many kilometers do you have to travel to get from home to HLH?

_______ KM

3E Date of injury, date of admission, duration of admission:

Date of injury:

____

Date of admission to HLH:

____

Delay from injury to admission:

____

Duration of admission:

____

4. SURGICAL CAPACITY

4A Healthcare received before admission to HLH:
What kind of treatment did you receive before you came to HLH?

☐None

☐Traditional healer

☐Dispensary

☐Primary healthcare facility

☐Hospital

Description of previous treatment

____

4A.2 Reasons for not having received surgical treatment before admission at HLH:
[Only when "none" is answered to 4A]
What was the main reason not to go to a health facility to see a doctor/nurse or not to have an operation or dressings?

☐No need

☐No money for health care

☐No (money for) transportation

☐No time

☐Fear/no trust

4A.1 How was the burn wound initially treated?
[Only when "dispensary", "primary healthcare facility" or "hospital" is answered to 4A]

☐Conservative treatment

☐Escharotomy

☐Escharectomy

☐Debridement

☐Skin grafting

☐Amputation

4A.1 Was there an indication for surgical care?

☐Yes

☐No

4B Did you receive surgical care at HLH?

☐Yes

☐No

4B.1 If yes, what kind of surgical care did you receive and how often?
[Only when "yes" is answered to 4B]

☐Contracture release

☐FTG

☐SSG

☐Z-plasty

☐Transposition flap

4B.2 If no, what were the reasons for not receiving surgical care?
[Only when "no" is answered to 4B]

☐No need

☐No money for health care

☐No (money for) transportation

☐No time

☐Fear/no trust

☐Not available (facility/personnel/equipment)

<u>5. AFFORDABILITY</u>

5A Insurance:
Do you have an insurance?

☐NHIF

☐CHF

☐Other

☐None

5B Daily budget
What is the daily budget of the household?

________ Tanzanian shilling per day

☐Tsh 0 – Tsh 4500 per day

☐Tsh 4500 – Tsh 6000 per day

☐Tsh 8000 – Tsh 10,000 per day

☐Tsh 10,000 – Tsh 15,000 per day

☐Tsh 15,000 – Tsh 25,000 per day

☐Tsh 25,000 – Tsh 50,000 per day

☐Tsh >50,000 per day

5C Amount of patient fee

Total amount of patient fee

________ Tanzanian shilling

% of patient fee covered by patients

________ %

% of patient fee covered by HLH

________ %

5D Transportation costs:

What does it cost you to travel one time from home to HLH?

________ Tanzanian shilling
